# Supplementary figures and images for: Full immunization coverage and associated factors among children aged 12–23 months in Somali Region, Eastern Ethiopia
Source: PLoS One. 2021 Dec 7;16(12):e0260258. doi: 10.1371/journal.pone.0260258 (PMC8651113; doi:10.1371/journal.pone.0260258)

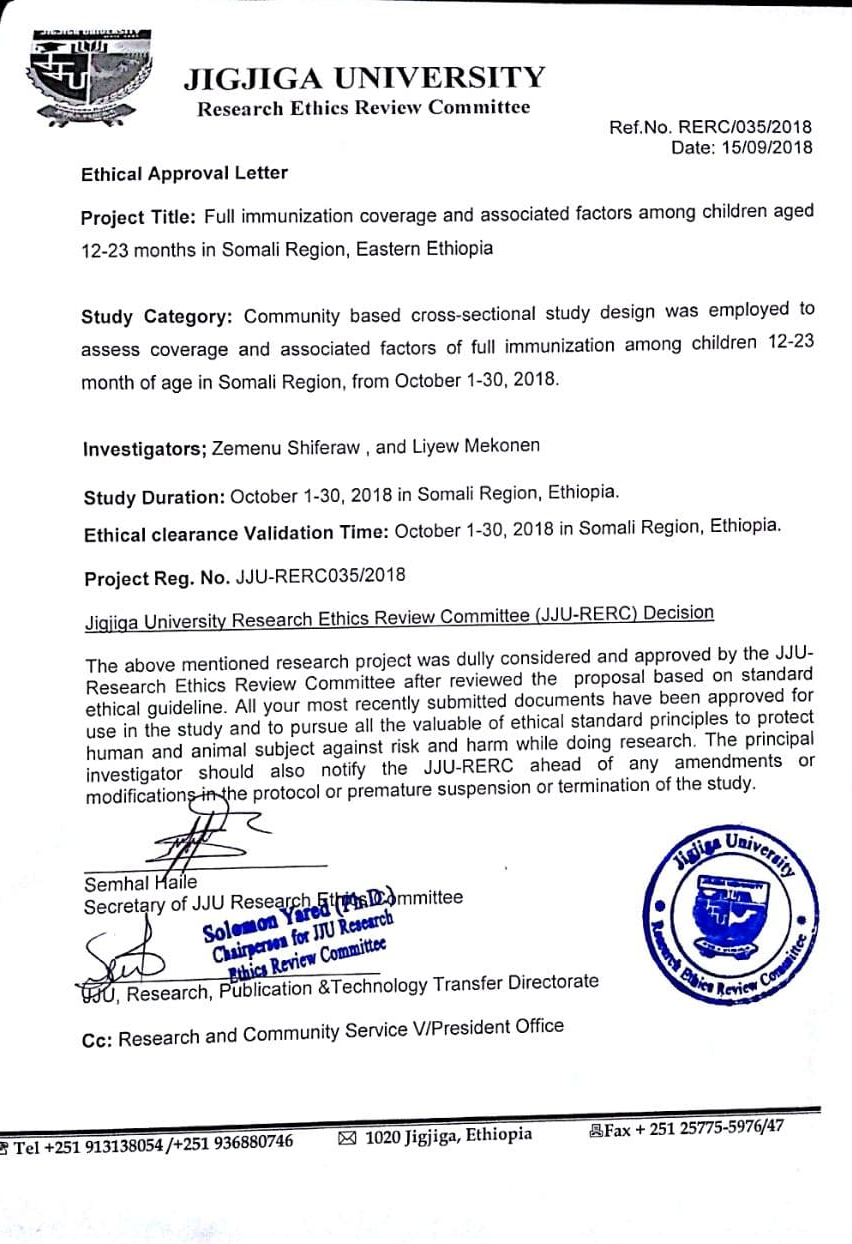

Supplement: S1 Fig — (TIF) [file pone.0260258.s001.tif]
