# Supplementary material for: Full immunization coverage and associated factors among children aged 12–23 months in Somali Region, Eastern Ethiopia
Source: PLoS One. 2021 Dec 7;16(12):e0260258. doi: 10.1371/journal.pone.0260258 (PMC8651113; doi:10.1371/journal.pone.0260258)
Supplement: S1 File — (PDF) [file pone.0260258.s002.pdf]

Annex

Annex I: Information Sheet

Good morning/afternoon, my name is ___________________. I am one of the data collectors for the study being conducted by Zemenu Shiferaw and Liyew Mekonen on the assessment of "**Full immunization coverage and associated factors among children aged 12-23 months in Somali region, Eastern Ethiopia"**

Title of the study: Full immunization coverage and associated factors among children aged 12-23 months in Somali region, Eastern Ethiopia".

Purpose: The purpose of the study was to collect information on **Full immunization coverage and associated factors among children aged 12-23 months.**

Procedure and Participation: The method of this study was a community based cross-sectional study and you are being selected to participate in this study by random sampling method. You will be asked about your socio-demographic and economic status, child characteristics, reproductive/obstetric history, accessibility of vaccination service (travel time), immunization histories of children, your knowledge on immunization, and reasons for defaulting. The expected duration of the participant's contact with the interviewer will be not more than thirty minutes.

Confidentiality: I assure you that all information collected from you will be confidential. To establish confidentiality of research data, the data collector will use codes during data collection period instead of using names. Only the researchers will have access to the information. No information will release to anyone outside of this data collection activity.

Benefit of the study: The research does not have a short term financial and health care benefit to the research participant as an individual or as a group but in the long term it will help the concerned organization and policy makers to have a policy consideration and direction and formulation of strategy based on the recommendations and the findings. In turn it will help the community indirectly through decreasing the neonatal morbidity and mortality. The result can be used as a source of information for further studies that can be done in this area.

Risk of the study: We do not anticipate that any harm will occur to you and your family as a result of participation in this study.

Right of the participant: Taking part in this study is completely your will. If you choose not to participate or if you decide to stop participating in this study, you will not get any harm and nobody will enforce you to explain the reason of withdrawal/refusal. You can stop participating in this study at any time even though you have already given your consent. Participant can skip question which the mother does not want to respond.

If you have questions about this study, you could contact principal investigators:

Zemenu Shiferaw:	Tel: +251910028125
Liyew Mekonen	Tel: +251921626447

Annex –II: Consent form

I have been told that this research is undertaken by Zemenu shiferaw and Liyew mekonen I have been fully informed in the language I understand and the objective of this research is to assess full immunization coverage and associated factors among children aged 12-23 months in Somali Region, Eastern Ethiopia.

I have been also informed that all the information I provide to the interviewer will be kept confidential. I understood that the research has no any risk. I also knew that I have the right to not answer the question that I don't want to answer or to withdraw from the study at any time I have acquainted nobody will enforce me to explain the reason of withdrawal.

I read this form, or it has been read to me in the language I understand and I understood the condition stated above.

1. Therefore, I am willing to participate ____________ (signature)
2. But, I am not willing to participate ______________ (tick)

Interviewer signature ___________________________ Date ______________________

Annex III: Data Collection Questionnaire (English Version)

	**Part- 1: socio- demographic characteristics and reproductive history**		
№:	Questions	Response	Skip
101	Residence	1. Urban<br>2. Rural	
102	Address of respondent?	Kebeles: ______________	
103	Age of the mother in years	__________________________	
104	Mother Religion?	1. Muslim<br>2. Orthodox<br>3. Protestant<br>4. Catholic<br>5. Other/ specify:___________.	
105	Mother Ethnicity?	1. Somali<br>2. Oromo<br>3. Amhara<br>4. Hareri<br>5. Other/ specify:________________.	
106	Maternal Marital status?	1. Single<br>2. Divorced<br>3. Married<br>4. Widowed<br>5. Other specify: _________________.	
107	Maternal educational status?	1. Illiterate<br>2. Read and write<br>3. Primary school (1-8)<br>4. Secondary school (9-12)<br>5. Above 12 (college/UN)	
108	Maternal occupation?	1. Housewife<br>2. Merchant<br>3. Government employed<br>4. Farmer<br>5. Other/ specify:______________.	
109	Family's average monthly income in birr?	_____________________________	
110	Age of the child in months	___________________	
111	Sex of the child	1. Male<br>2. Female	
112	Number of children's older siblings	_______________________	
113	Family size	____________	

114	How many living children do you have now?	Male: ________<br>Female: _______.	
115	Have you attended antenatal care during your last pregnancy?	1= Yes<br>2= No	If no skip to 1.17
116	If yes, how many times did you attend?	________________	
117	Have you received tetanus vaccination during your last pregnancy?	1. Yes<br>2. No	If no skip to 1.19
118	If yes, how many injections did you received?	_____________	
119	Where did you deliver your last baby?	1. at home<br>2. at health institution<br>3. Other___________________	
120	Birth order of the current birth?	1. First<br>2. Second<br>3. Third<br>4. Fourth & Above	
	**Part 2: Questions to assess access to Immunization, and related knowledge**		
No:	Question	Response	Skip
201	Is there any health facility which vaccination service near to you?	1. Yes<br>2. No	If no skip to 2.7
202	If yes to above question which health facility is near to you?	1. Health center<br>2. Hospital<br>3. Health post<br>4. Private clinic	
203	How long it take you to reach there in minutes?	1. Less than 15 minutes<br>2. 15-30 minutes<br>3. 30-1hour minute<br>4. > 1 hour	
204	When you reach at the health facility How many minutes it takes for you to wait at the health facility to get vaccination?	1. Less than 15 minutes<br>2. 15-30 minutes<br>3. > 30 minute	
205	Is there functional vaccine Refrigerator (Observe)	1. Yes<br>2. No	
206	Is there defaulter tracing mechanism (Observe)	1. Yes<br>2. No	

207	Do you heard about vaccination and vaccine preventable disease?	1. Yes<br>2. No	
208	If yes to above question, from where do you heard about the vaccination and vaccine preventable disease?	1. Radio<br>2. Television<br>3. Health Personnel<br>4. From Friends/Peers<br>5. Community education Program<br>6. Other, Specify ____________	
209	Could you mention the objective of vaccinating a child?	1. To prevent the disease<br>2. For specific disease<br>3. For child health<br>4. Don't know<br>5. Other, specify ______________	
210	How many vaccine preventable diseases do you know? _____________	1. Measles<br>2. Tetanus<br>3. Pertusis<br>4. Tuberculosis<br>5. Diphtheria<br>6. Polio<br>7. Hepatitis B infection<br>8. Homophiles influenza b<br>9. Rota virus infection	
211	How many vaccination sessions are needed for a child to be fully protected?	1. One<br>2. Two<br>3. Three<br>4. Four<br>5. Five and more<br>6. don't know ________________	
212	Can you tell me the age at which the child begins immunization?	1. Just after birth<br>2. One month after a birth<br>3. Any time<br>4. After one year<br>5. I don' know<br>6. Other specify_______________	
213	At what age the child should complete immunization?	_______________	
	**Part 3: Question to assess Immunization status**		
No	Question	Response	Skip
301	Does your child take any vaccination?	1. Yes<br>2. No	

302	Do you have a card where vaccinations are written down?	1. Yes<br>2. No		If no skip to 3.4
303	If Yes, copy the immunization data from the card, as follow			
	**Vaccine taken**	**Date**	**Month**	**year**
	BCG & OPV0	-----------	-----------	-----------
	OPV1	-----------	-----------	-----------
	OPV2	-----------	-----------	-----------
	OPV3, IPV	-----------	-----------	-----------
	Pentavalent1, PCV1, Rota1	-----------	-----------	-----------
	Pentavalent2, PCV2, Rota2	-----------	-----------	-----------
	Pentavalent3, PCV3	-----------	-----------	-----------
	Measles, Vitamin A	-----------	-----------	-----------

304	Would tell me if the child had any of the following vaccinations (vaccination by history)		
	a) A BCG vaccination against tuberculosis, that is, an injection in the arm or shoulder that usually causes a scar	1. Yes____<br>2. No______	
	b) Polio vaccine, that is, drops in the mouth?	1.Yes__________<br>2. No_____________	
	c) Was the first polio vaccine given in the first two weeks after birth or later?	Yes__________<br>No_____________	
	d) How many times was the polio vaccine given	______________	
	e) A pentavalent vaccination, that is, an injection given in the thigh or buttocks?	Yes__________<br>No_____________	
	f) How many times Pentavalent vaccination was given?	________________	
	g) A measles injection that is, a shot in the arm at the age of 9 months or older – to prevent him/her from getting measles?	Yes__________<br>No_____________	
	h) Does the child have a BCG scare on his/her upper left arm? Observe	Yes__ No ___	
	i) Rota virus vaccine, that is, drops in the mouth?	Yes__ No ___	
	j) How many times was Rota vaccine given	_______________	

	k) A PCV (Pneumococcus vaccine) vaccination, that is, an injection given in the thigh	Yes__ No ___	
	l) How many times was PCV given?	________	
305	**If the child defaulted ask the following**		
	What are the reasons for defaulting? (If child is a defaulter)	1.Vaccination site is far-away<br>2. Vaccination time is inconvenient<br>3. Absenteeism of vaccinators<br>4. Lack of awareness on the importance of vaccination<br>5. Not knowing vaccination time and site<br>6. Not knowing whether to come back for second and third vaccination<br>7. Others	

THANKS!

Annex III: Data Collection Questionnaire (Somali Version):

	**Part- 1: xogta bulsho iyo xaalada taranka**		
Nº:	Suaalaha	jawaabaha	Dhaaf
1.1.	Degananshaha	3. Magaalo<br>4. Miyiga	
1.2.	Ciwaanka ka qayb qaataha	Kebeles: ______________	
1.3.	Da'da hooyada sanadaha	__________________________	
1.4.	Diinta Hooyada	6. Muslim<br>7. Orthodox<br>8. Protestant<br>9. Catholic<br>10. Midkale/ sheeg:___________.	
1.5.	Isirka Hooyo	6. Somali<br>7. Oromo<br>8. Amhara<br>9. Hareri<br>10. Mid kale/ sheeg________________.	
1.6.	Xaaladda guurka hooyada?	6. Kalidaa<br>7. La Furray<br>8. Guursaday<br>9. Carmallay<br>10. Wax kale sheeg _________________.	
1.7.	Heerka waxbarashada hooyada	6. Waxna qorin<br>7. Akhriya oo qora<br>8. Dugsiga hoose (1-8)<br>9. Dugsiga sare (9-12)	

		10. Ka sarreeya 12 (kulliyad/UN)	
1.8.	Shaqada hooyada?	6. Xaaska -guriga<br>7. Ganacsato<br>8. Dawlada oo shaqaysa<br>9. Beeraley<br>10. Mid kale/ sheeg: ______________.	
1.9.	Celceliska dakhliga soo gala bishii qoyska ee birta?	______________________________	
1.10.	Da'da ilmaha bil ahaan	____________________	
1.11.	Jinsiga ilmaha	3. Lab<br>4. Dheddig	
1.12.	Tirada carruurta walaalo yihiin ee ka weyn	________________________	
1.13.	Tirada qoyska	_____________	
1.14.	Imisa carruur ah ayaad hadda haysataa?	lab: _________<br>Dhadig: _______.	
1.15.	Miyaad qaadatay daryeelka dhalmada kahor uurkaagii u dambeeya?	1= Haa<br>2= Maya	hadii ay maya tahay u bood 1.17
1.16.	Hadday haa tahay, imisa jeer ayaad tagtay?	________________	
1.17.	Miyaad qaadatay tallaalka teetanada inta aad uurka leedahay?	1. Haa<br>2. Maya	hadii ay maya tahay u bood 1.19
1.18.	Hadday haa tahay, imisa irbadood ayaad qaadatay?	_____________	
1.19.	Xaggee baad ku umushay ilmahaagii ugu dambeeyay?	1. guriga<br>2. xarunta caafimaadka	

		3. . Mid kale___________	
1.20.	Imahani waa ilmiihi imisaad ciyaalkaga?	1. kowaad<br>2. kii labaad<br>3. Kii sSaddexaad<br>4. Afraad & Kor	
	**2. Part 2: Su'aalaha lagu qiimeynayo helitaanka Tallaalka, iyo Macluumaadka la xiriira**		
No:	Suaalaha	Jawaabaha	Dhaaf
2.1.	Ma jiraa xarun caafimaad oo adeegga tallaalka kuu dhow?	3. Haa<br>4. Maya	hadii ay maya tahay u bood 2.7
2.2.	Hadday haa tahay su’aasha kore xarun caafimaad Tee kuugu dhow?	5. Xarunta caafimaadka<br>6. . Isbitaal<br>7. . Rug caafimaadka<br>8. Rug caafimaad oo gaar loo leeyahay	
2.3.	Intee in le'eg ayay kugu qaadanaysaa inaad gaadho xarunta cafimaadka daqiiqado?	5. In ka yar 15 daqiiqo<br>6. 15-30 daqiiqo<br>7. 30-1saacadood<br>8. .> 1 saac	
2.4.	Markaad gaarto xarunta caafimaadka Immisa daqiiqo ayay kugu qaadanaysaa inaad ku sugto xarunta caafimaadka si aad u hesho tallaal?	4. In ka yar 15 daqiiqo<br>5. 15-30 daqiiqo<br>6. > 30 daqiiqo	
2.5.	Ma jirtaa tallaajada talalka oo shaqeynaysa (U fiirso)	3. Haa<br>4. Maya	
2.6.	Ma jiraa farsamo lagu daba galo ciyaalka kala gooya talaalka (U fiirso)	3. Haa<br>4. Maya	

2.7.	Miyaad Maqashay tallaalka iyo cudurada tallaalka lagaga hortagi karo?	3. Haa<br>4. Maya	
2.8.	Hadday haa tahay su'aasha kore, xaggee baad ka maqashay tallaalka iyo cudurada laga hortagi karo tallaalka?	7. Raadiyaha<br>8. Telefishanka<br>9. Shaqaalaha Caafimaadka<br>10. Ka yimid Asxaabta/Asxaabta<br>11. .Barnaamijka waxbarashada bulshada<br>12. Mid kale, Sheeg	
2.9.	Miyaad sheegi kartaa ujeeddada tallaalka loo siinayo ilmaha?	6. looga hortago cudurka<br>7. Cudur gaar ah<br>8. Caafimaadka ilmaha<br>9. Ma ogi<br>10. Mid kale, sheeg _______________	
2.10.	Immisa cudur oo laga hortagi karo tallaalka ayaad taqaan? ______________	10. jadeecad<br>11. Tetanada ama Daxalka<br>12. Xiiq dheer<br>13. Qaaxada<br>14. Qawracato<br>15. Dabayl<br>16. Joonis<br>17. Hargab<br>18. Shuban	
2.11.	Imisa kulan oo tallaal ayaa loo baahan yahay si ilmuhu u noqdo mid si buuxda uga badbaday xanunada talaal la’antu keeno?	7. Hal mar<br>8. Laba jeer<br>9. Sadex jeer<br>10. Afar jeer<br>11. Shanjeer iyo in ka badan<br>12. Magaranayo _________________	

<table>
<tr><td>2.12.</td><td>Ma ii sheegi kartaa da'da uu ilmuhu bilaabo tallaalka?</td><td>7. Dhalashada kadib
8. . Bil kadib dhalashada
9. . Wakhti kasta
10. . Hal sano kadib
11. . Ma aqaan
12. . Caddayn kale ________________</td><td></td></tr>
<tr><td>2.13.</td><td>Da'dee ayuu ilmuhu ku dhammaystirayaa tallaalka?</td><td>________________</td><td></td></tr>
<tr><td></td><td colspan="3">3. Part 3: Su'aalaha si loo qiimeeyo heerka tallaalka</td></tr>
<tr><td>Nº</td><td>Su.aalaha</td><td>Jawabaha</td><td>Dhaaf</td></tr>
<tr><td>3.1.</td><td>lmahaagu ma qaatay wax tallaal ah?</td><td>3. Haa
4. Maya</td><td></td></tr>
<tr><td>3.2.</td><td>Miyaad leedahay kaar lagu qoray tallaalada?</td><td>3. Haa
4. Maya</td><td>Hadii ay maya tahay u bood 3.4</td></tr>
<tr><td>3.3.</td><td colspan="3">Hadii ay haa tahay ku qor xagan xogta talalka ee karaka ku qoran sidan soo socota
<table>
<tr><th>Nooca talaalka u qaatay</th><th>Maalinta</th><th>Bisha</th><th>Sanadka</th></tr>
<tr><td>BCG & OPV0</td><td></td><td></td><td></td></tr>
<tr><td>OPV1,</td><td></td><td></td><td></td></tr>
<tr><td>OPV2,</td><td></td><td></td><td></td></tr>
<tr><td>OPV3, IPV</td><td></td><td></td><td></td></tr>
<tr><td>Pentavalent1, PCV1, Rota1</td><td></td><td></td><td></td></tr>
<tr><td>Pentavalent2, PCV2, Rota2</td><td></td><td></td><td></td></tr>
<tr><td>Pentavalent3, PCV3</td><td></td><td></td><td></td></tr>
<tr><td>Measles, Vitamin A</td><td></td><td></td><td></td></tr>
</table></td></tr>
</table>

3.4.	Ma isheegi kartaa haddii ilmuhu lahaa mid ka mid ah tallaalada soo socda (tallaalka taariikhda)		
	m) Tallaalka BCG ee ka hortagga Qaaxada, taas oo ah, cirbad gacanta ama garabka oo badiyaa sababa nabar	3. Haa____<br>4. Maya______	
	n) Tallaalka dabaysha, oo ah, dhibcaha afka?	1.Haa__________<br>2. Maya_____________	
	o) Tallaalka dabaysha ee ugu horreeya ma la siiyay labadii toddobaad ee ugu horreeyey kadib dhalashada ama ka dib?	1.Haa__________<br>2.Maya_____________	
	p) Imisa jeer ayaa la siiyay tallaalka dabaysha	______________	
	q) Tallaalka pentada oo ah, cirbad lagu siiyay bowdada ama badhida?	1.Haa__________<br>2. Maya_____________	
	r) Imisa jeer ayaa la siiyay tallaalka Pentavalent?	________________	
	s) Cirbadda jadeecada oo ah, xabbad gacanta ah markay tahay 9 bilood ama ka weyn - si looga hortago in isaga/iyada uu ku dhaco jadeeco?	Haa__________<br>Maya_____________	
	t) lmuhu ma leeyahay boogta BCG gacanta/bidix ee kore? U fiirso	Haa__ Maya ___	
	u) Tallaalka fayraska Rota, oo lagaga hortago shubanka oo ah, dhibic afka?	Haa__ Maya ___	
	v) Imisa jeer ayaa la siiyay tallaalka Rota lagag hortago shubanka	_______________	
	w) Tallaalka PCV (tallaalka Pneumococcus),oo lagaga hortago oofwareenka taas oo ah, cirbad laga siiyay bowdada	Haa __ Maya ___	
	x) Imisa jeer ayaa la siyay tallalka PCV ee oofwareenka ?	________	
	**Haddii ilmuhu kala gooyay tallalka waydii kuwan soo socda**		

3.5.	Waa maxay sababaha uu ukala gooyay talalka ? (hadii uu ilmuhu kala gooyay)	8. Goobta tallaalku waa meel fog<br>9. Waqtiga tallaalku waa mid aan habboonayn<br>10. Maqnaanshaha tallaalayaasha<br>11. Wacyigelin la’aanta ahmiyadda tallaalka<br>12. Ma garanayo waqtiga tallaalka iyo goobta<br>13. Ma garanayo inaad ku soo noqonayo tallaalka mar labaad iyo saddexaad<br>14. Kuwo kale	

Wad mahansid!
