# Supplementary material for: Full immunization coverage and associated factors among children aged 12–23 months in Somali Region, Eastern Ethiopia
Source: PLoS One. 2021 Dec 7;16(12):e0260258. doi: 10.1371/journal.pone.0260258 (PMC8651113; doi:10.1371/journal.pone.0260258)
Supplement: Attachment — Submitted filename: Response to reviewers (2).docx [file pone.0260258.s004.docx]

# **Response to reviewers**

I. **Comments from Academic Editor and Response to the Academic Editor:**
1. ***Comments:*** *Please ensure that your manuscript meets PLOS ONE's style requirements, including those for file naming.*

**Response:** Maximum effort has been made to make sure that our manuscript meets PLOS ONE's style requirements.

2. ***Comments:*** *Please include additional information regarding the survey or questionnaire used in the study and ensure that you have provided sufficient details that others could replicate the analyses. For instance, if you developed a questionnaire as part of this study and it is not under a copyright more restrictive than CC-BY, please include a copy, in both the original language and English, as Supporting Information.*

**Response:** Information’s regarding the questionnaire is provided in the method section. In addition, the questionnaire is submitted as a supporting information in English and Somali language.

3. ***Comments:*** *In the Methods, please clarify that participants provided oral consent. Please also state in the Methods:
   - Why written consent could not be obtained
   - Whether the Institutional Review Board (IRB) approved use of oral consent
   - How oral consent was documented
   - Whether consent was obtained from the guardians/parents of minors*

**Response:** Both, oral consent and written consent was taken from the mothers/caregivers of children aged 12-23 months. This information’s are included in the method section.

4. ***Comments:*** *We note that you have indicated that data from this study are available upon request. PLOS only allows data to be available upon request if there are legal or ethical restrictions on sharing data publicly.*

**Response:** All data supporting the findings is submitted with the manuscript. The data set for this article is openly accessible without restriction. The data set is submitted as supporting information file as SPSS version 23 data set.

II. **Response to the reviewer #1**

**Comments:** This is a great paper, I have enjoyed reading it.
Authors are encouraged to read through the manuscript for any typographic errors. More specifically on page 15; line 203 where figure 1 is indicated twice.

**Responses:**

1. Maximum efforts have been made to make sure the data supports the conclusion.
2. Both descriptive and analytical statistical analysis were performed thoroughly.
3. All data supporting the findings is submitted with the manuscript. The data set for this article is openly accessible without restriction.
4. The manuscript is written in standard English. Typographic errors, and grammatical issues have been corrected.
III. **Response to the reviewer #2**
I. Abstract:
   1. ***Comment:*** *Better to adhere the guide line of PLoS one journal. All major sections is needed*
      **Response:** The abstract is developed in line with PLoS one journal guide line.
   2. ***Comments:*** *The knowledge gap is not well addressed that needs to be explained more at abstract section.*

**Response:** The knowledge gap, the statistical models and measurements are clearly presented in the abstract.

3. ***Comments:*** *Word consistency “associated factors or predictor factors”*
   **Response:** Corrected
4. ***Comments:*** *At method section, say something about measurement and type of model for different outcome interest of the study.*

**Response:** the measurements and statistical models have been clearly presented.

5. ***Comments:*** *At result section, result should be stick to the objective of the study.*

**Response:** maximum effort has been made to stick with the objectives of the study.

II. Introduction:
    1. ***Comments:*** *Authors should briefly explain what has been done so far. To be more interesting, author need to consider “what is the additional knowledge has is this study going to generate”? The knowledge gap is not well addressed in the introduction of the manuscript and needs to be explained more.*

**Responses:** It clearly shows the knowledge gap. In Ethiopia, progresses on child immunization has been made; however, the full immunization coverage is below the national and global targets. On the other hand, evidence on full immunization and associated factors are very scarce in the hard to reach regions of Ethiopia, particularly in Somali region.

2. ***Comments:*** *Explain why more work/research is necessary. What contribution to knowledge that the research will make and its place in current debate or technological advances?*

**Responses:** The introduction clearly presented assessment of full immunization and associated factors would be a new knowledge for the study area. Hence, this study will serve as an important evidence on child full immunization in the Somali region.

3. ***Comments:*** *Some references are olds, needs to be justify the research question or problem by using current evidences.*

**Responses:** Some of the old references were used to define concepts; but not to justify the problem gap.

4. ***Comments:*** *There are some studies conducted in the study area, what additional knowledge gap is addressed by this study.*

**Response:** There are some studies on child immunization in the study area. But, they didn’t address the full immunization coverage and associated factors.

5. ***Comments:*** *A grammatical and linguistic edit is essential for this manuscript, as the numerous issues are apparent in it and make understanding the provided draft difficult.*

**Responses:** Grammatical errors are corrected.

6. **Comments:** What is the objective of the study? Word consistency, at introduction section assess prevalence and factors, at abstract section “Identifying predictor factors, assessed full-immunization and associated factors, at method part “assess coverage and associated factors of full immunization”----these all makes confusion for the reader.

**Responses:** The objective of the study was presented in different ways but with similar concept. Corrections has been made accordingly.

III. Method and materials:
     1. ***Comments:*** *A Method part looks like master thesis protocol. Concepts should be explained based on the guideline of the journal.*

**Response:** The concepts in the method section were explained based on the guideline of the journal.

2. ***Comments:*** *Authors said “study populations were randomly selected children aged 12-23 months with their mothers/caregivers residing in the Somali region”. How randomly select the study population? What random method means?*

**Responses:** Probability/random sampling methods were consistently used to select study participant.

3. ***Comments:*** *What are the inclusion and exclusion criteria.*

**Responses:** Inclusion and exclusion criteria for study participants are included in method section. “**Inclusion Criteria:** Mothers with at least one child aged between 12-23 months who did take at least one dose of any vaccine were included. **Exclusion criteria:** Those mothers who were unable to respond or very sick were excluded.”

4. ***Comments:*** *At sampling technique and procedure section important information is missed, like total number of kebeles in the district, total number of the households in the selected kebeles, Authors apply systematic random sampling method, how to apply important step/information/ is not documented.*

**Responses:** Accepted and corrected

5. ***Comments:*** *If authors conduct pretest, what was the modified things, you should be report and document in the manuscript*

**Response:** Pretest was done on the questionnaire but major modifications were not found.

6. ***Comments:*** *Your tool is adopted from DHS and another literature with some modification, so it needs tool validation, how to check reliability and validity of the tool? Cite the source of tools? The procedure of data collection?*

**Responses:** The content validity of the questionnaire was achieved by reviewing the previous similar studies. Data collection procedure were clearly and precisely presented in the data collection section.

7. ***Comments:*** *What are unique variables/factors/ examined compared to the pervious available studies. All identified variables are already addressed previously studies. therefore, include variable should not be redundant.*

**Responses:** Most variable were not addressed in the study area. Previously conducted studies addressed variables related to child immunization, while this study addressed major variables related to “child Full-immunization”. These variables include; socio-demographic and economic status, child characteristics, reproductive/obstetric history, accessibility of vaccination service (travel time), immunization histories of children, maternal knowledge on immunization, and reasons for defaulting.

8. ***Comments:*** *In the measurement section, what is the reference for the standardized tool for full immunization measurement? Also, please add whether the tool is standardized for the Ethiopian population or not. How many knowledge questions are asked? How the questions are designed?*

**Responses:** Accepted and corrected.

9. ***Comments:*** *Statistical analysis, replace by data processing and analysis important information are lacking like Checking of assumption (Normality and interaction effect).*

**Responses:** Data processing and analysis issues were clearly and precisely presented. For instance, Chi-square testing were used and normality were checked. Binary logistic regression model was employed to identify associated factors. Variance inflation factor (VIF) was checked to see interaction effects (Multi-collinearity) between the variables.

10. ***Comments:*** *Who approve the study? How get consent? Ethical consideration is the major concern today*

**Responses**: The study was approved by Jigjiga University Ethical Review Board. Written consent was sought from study participants before data collection. Participants were verbally informed about the objective of the study, confidentiality of their data and the right to refuse participation.

IV. Results
    1. **Comments:** Results did not provide new knowledge in this field.

**Response:** Every finding in the result section is new, because it is very rare to find evidence on full immunization coverage and associated factors, in the pastoralist and Agro-pastoralist regions of Ethiopia and Africa.

2. **Comments:** In the regression table, some of the results indicates wide confidence interval, are you trusting these findings?

**Response:** Some of the results in the regression table showed wider confidence intervals because of smaller cell values. However, since assumptions were checked for binary logistic regression analysis, still the findings are trusted.

V. Discussion:
   1. ***Comments:*** *It is quite poor and repeats a lot of known facts without making any point as to how this current study contributes. A lot of results are repeated in the discussion. What are the innovative ideas, for scale up and ensure quality and safe services? Formulate clear what is innovating idea in the study.*

**Responses:** The discussion clearly presented the realities in the one of pastoralist region of Ethiopia compared to national and global literatures. This study will be an input for local and regional quality improvement programs.

2. ***Comments:*** *Your results may be affected by social desirability bias because questionnaires were collected by interviewers; the participants were unable to remain anonymous. This should be mentioned in the limitations section.*

**Responses:** Accepted and corrected.

VI. Conclusion:
    1. **Comments:** Conclusion is repeated, there is significant disconnect between the results presented and the conclusions made. There was no evidence in the results or anywhere else that they looked at the possible barriers and strategies for that country under question. They can suggest but not make a hard conclusion that those strategies would work or hinder.

**Responses:** All conclusion has been made based on the findings in the result.

2. **Comments:** Recommendation-This paper is below the scope of the journal, so consider after major changes

**Responses:** Recommendations were suggested based on the conclusions.
